# Some Reasons Why We Fail with Cataphoresis

**Published:** 1897-02

**Authors:** W. H. Hersh

**Affiliations:** Piqua, Ohio


### Some Reasons why we Fail with Cataphoresis.

BY W. H. HERSH, PIQUA, OHIO.

*Mr. President and Gentlemen:*

There has been no subject during the past year that has received as much study, experimenting with, writing upon and discussing, as the one commonly known as "Cataphoresis." No one theme has received as much time in our various societies, or been given as much space in our dental periodicals, or excited such universal interest among the members of our profession as the one to which we have just referred.

It shows conclusively that the dentist has been looking forward with no small degree of anxiety to the time when he should have an *ideal* obtundent, and anything that promises this is welcomed by all.

The first paper written upon this subject, during the present year, was prepared by a man who had carefully tested the merits of the method, and had apparatus especially made for the control of the current; one who had given up breadth of subject in order to gain depth, whose energies were all directed in the line of the relief of sensitive dentine by electrical osmosis. And when the results of this careful and skillful dentist, who had spent many months in this single branch of dental electro-therapeutics, were made known we were led to believe that it was an *easy* method and very closely and probably fully met that desire for the ideal obtundent for which we have been so long seeking. It was the kindling of the fire that has swept over the entire dental world.

My love for this peculiar power or energy led me early to test, investigate and experiment with this process, and with various appliances and combinations, until I am now satisfied that there is much to be done yet before it will fully meet our wants, and have found, as every one else has found having experience along this line, that it is *not* the panacea for all dental ills and so shall try and give "some reasons why we fail with cataphoresis," stating first that it is an established method, and one in which the possibilities seem practically unlimited.

First and foremost, is the reason that the average dentist is not prepared for this class of work. A physician who is skilled in the use of electricity as a remedial agent gives this advice to those who contemplate the use of electricity in their practice:

"First, make yourself a skilled physician, then study the physics, physiology, mechanics and chemistry of electricity; seek practical instruction in the technique of applying the same, then buy your outfit."

How reversely true is that of the dentist who wishes to use electricity as a therapeutic agent, or wishes to use it to cause deeper penetration of other agents. Does he not first buy his outfit then seek instruction in the technique of applying it from a circular of a few pages? If success attends his efforts he will probably look deeper into the subject, but if not successful will abandon the apparatus and method and call them worthless, when he, himself, is the worthless part of the outfit. It is puzzling to me that men should even *expect* success when handling an agent they know so little about, in many instances being doubtful that there is any virtue in the method, probably not knowing that plus (+) stands for positive or minus (—) negative, or what the difference between the two is, having but little idea of what the simpler terms mean, or the action of the current in any but its most common forms. Is it then not reasonable to believe that a method that requires such skill in handling and nicety of adjustment in order to be a success should be a *failure* in the hands of careless operators, or those not understanding their apparatus thoroughly, or what is going on between the two electrodes. And so it seems fitting to put the operator first in the reasons why we fail with cataphoresis, believing that a better general knowledge of elementary principles of Galvanism will be necessary before the method can become the universal obtundent.

The next reason in importance as to why we fail, is the source of the current supply, and, in fact, it seems almost as necessary that we have a smooth, even current as that we have a skilled operator to apply it. No doubt, among the better class of operators, no one factor has played such an important part in their failures as an irregular and unreliable current excepting, as a matter of course, an inferior apparatus.

Let me first say that, in my opinion, the 110-volt commercial current should *never* be used in cataphoric procedures. This statement is not made on any theoretical grounds, but by actual and persistent experience in the use of the 110-volt current in this work.

We found, first, that it was *not* the current for use in the relief of sensitive dentine and so discarded it for that, using cells, but retained it for bleaching and the other work; in fact, everything except the obtunding of sensitive dentine, but later on discarded it, giving cells the preference even in this class of work. My reasons for making the statement that it should never be used is based on several factors, viz.: First, because it is a *dangerous* current to use in this extremely-sensitive and delicate work. Dangerous because of a possible ground through the fountain spittoon, an experience which your humble servant has had, and one which he will never take the risk of having repeated; dangerous because of atmospheric electricity, or lightning, coming in contact with your mains; dangerous because of wires carrying powerful currents coming in contact with the system; dangerous because of the possibilities of having a lamp burn out or broken, or a connection becoming loose, and many other things which might cause it to prove disastrous, make it a current that we should not accept the risks of using in this work about the head, *especially* when a satisfactory current can be so cheaply obtained from a few dry cells, and one which will answer every purpose and require no care on the part of the operator.

Second, because in the majority of cities the current fluctuates and is too irregular to be used in this work. The sudden raising or lowering of the voltage, which is unavoidable in most plants, will cause pain and failure.

Third, because when we have put enough resistance in the path of the current to reduce it so we can use it, we have a current of high electro-motive force, but with very little volume, while with the cell we have just the opposite condition of affairs. To verify this statement we will give you two tests made that will more clearly bring this fact out, viz.: Three mesco dry cells in series gave 4.3 volts. The 110-volt current was then shunted until we had as a maximum voltage 8 volts and reduced to 4.3 volts by a graphite resistance, on short circuit we found the 110-volt current to give as its maximum strength 0.8 of a milliampere, while the three cells gave 5.5 amperes, or 5,500 milliamperes, the 110 volt less than one milliampere, and the cells 5,500 milliamperes. The second test was with 6 mesco cells in series which gave 8.3 volts and 5.5 amperes, or 5,500 milliamperes, while the 110 volts shunted to 16 volts, and reduced by the graphite rheostat gave 6 8 milliamperes.

We would not have you understand by this that a certain voltage generated by cells has more power to overcome resistance than the same number of volts derived from any other source, even though the ampereage or volume be much larger, but this fact I would have you note, that resistance reduces the amperage or volume much faster than it does the voltage or electromotive force, and further, that men in the medical profession who are intimately acquainted with the physical laws of electricity, as well as those having years of experience in the use of electricity in the treatment of disease, have found that the least number of cells that would furnish the desired amount of current without the intervention of a rheostat, or external resistance of *any* kind, will produce the same results in the same time and with *less* pain than can be done with a larger number of cells and the voltage reduced by means of the rheostat. That being true in medicine it must be true in dentistry.

Fourth, because when the current is smooth and the voltage steady and even, we have a current which is not any *superior* to the cell, but to me very much *inferior* because of the attending dangers and the extra caution necessary in handling it. The cell is such an inexpensive thing that this can scarcely be urged as against it and there is such a small quantity used from it, it will, no doubt, do service for many months and will be found to be just as efficient in the office of the country practitioner as in the splendidly-equipped parlors of our city brethren.

Another reason that may cause failure, is because of too small negative surface. This fact must be remembered : the *larger* the negative electrode, the *less* the resistance, as the tooth operated upon and the skin form the major part of the resistance, and if too small an electrode is used it causes a burning or prickling sensation, and I have noticed in many cases a deep reddening of the skin, even though a very small amount of current was used. It also requires more current or voltage to get the same results, and, therefore, is more painful, which causes the patient to lose confidence and you stop short of success, while a liberal negative surface would materially lessen the resistance; therefore, require less electro-motive force and less pain to get the same results. A single experiment will serve to illustrate this. Three cells, using a negative electrode 1.5 inches in diameter and well moistened, gave us three-tenths of a milliampere, while a large negative sponge electrode gave, with the same number of cells, and through the same resistance, seven-tenths of a milliampere, which was more than double that of the first.

Another reason why our operators are not more uniformly successful is because of the lack of proper measuring instruments. It seems as if uniform results could hardly be expected in the administration of the current unless we knew just the amount of current that is actually passing through the tooth or soft tissues of the patient. It is my firm conviction that no one ought to administer the current to a patient without knowing precisely the amount being used. True it is that we can get along without it, but is it the *best* way to do and should we be hindered in any way by not having everything needed to give the best results?

Another thing that is the cause of such pain, annoyance and failure is the mistake many operators make by trying to hold the positive electrode in the tooth. This should be avoided whenever possible, as the least movement of the point in the tooth causes pain which sometimes makes the patient nervous and fretful, and you and the patient are both willing to quit before any deep obtunding effects are secured. A very convenient way to hold the electrode is to solder a piece of fine platinum wire on to the tip of the conducting cord and fusing a little ball on the point with the electric current so that when placed in the cavity and cotton packed around it, it will be retained in position, and thus save the operator the trouble and fatigue of holding it. The wire can be insulated by a small piece of fine regulating tubing. The time required in some cases which most need this method is a barrier to success that can not be lost sight of. The preparation of the cavity before commencing is a very important item, and the thorough insulation of the tooth and fillings is certainly one of the very necessary things to do, and if close attention is not given to this it will cause you to have a failure where otherwise you would be successful. Many other things might cause a failure, such as loose connection, inferior cords, corroded surfaces, stale solutions, inferior apparatus and many small things in detail which only experience can teach.

Please bear with me while we administer, we hope cataphoretically, a few words of advice. First, study some of the elementary principles of Galvanism, buy your outfit of a person or company who has thoroughly tested it and proved it to be reliable, then, for your first case, select one of the patients that you would dearly love to see leave you, and commence to use the cataphoric method, reserving the patients you care to keep until sufficient experience and confidence is gained to make it a practical obtundent; use a good apparatus, large negative electrodes, fresh solutions, and some judgment mixed in with the rest, and you will find in a little time that you have an obtundent which will make possible thorough operations, where before it was necessary to make temporary fillings, and your patients will thank you for your skill in handling this agent, and all the while you will be gaining experience and mastery over a process which promises a wider range of usefulness than any method brought to the notice of our profession during the last decade.

